# Thermal refugia and persistence of Texas horned lizards (*Phrynosoma cornutum*) in small towns

**DOI:** 10.1002/ece3.10245

**Published:** 2023-07-08

**Authors:** Mary R. Tucker, Daniella Biffi, Dean A. Williams

**Affiliations:** ^1^ Department of Biology Texas Christian University Fort Worth Texas USA; ^2^ Andrews Institute of Mathematics & Science Education Texas Christian University Fort Worth Texas USA

**Keywords:** microhabitat, shrubs, thermal ecology, thermoregulation, urban ecology

## Abstract

Vegetation loss is a primary cause of habitat degradation and results in a decline in reptile species abundance due to loss of refuge from predators and hot temperatures, and foraging opportunities. Texas horned lizards (*Phrynosoma cornutum*) have disappeared from many areas in Texas, especially from urbanized areas, probably in large part due to loss of suitable habitat. This species still occurs in some small towns in Texas that still contain suitable habitat. Long‐term data from Kenedy and Karnes City, Texas indicate that when study sites experienced significant shrub and vegetation removal horned lizards declined by 79%. We hypothesize the decline was due to the degradation of the thermal landscape for these lizards. We determined the preferred temperature range (*T*_set25_ – *T*_set75_) of lizards at our study sites and took field measurements of body temperature (*T*_*b*_). Temperature loggers were also placed in three microhabitats across our study sites. Shrubs and vegetation provided the highest quality thermal environment, especially for about 5 h midday when temperatures in the open and buried under the surface in the open exceeded the lizards' critical maximum temperature (CT_max_) or were above their preferred temperature range. Horned lizard density was positively related to the thermal quality of the habitat across our sites. Texas horned lizards in these towns require a heterogeneous mix of closely spaced microhabitats and especially thermal refugia, such as shrubs and vegetation along fence lines and in open fields. Maintaining thermal refugia is one of the most important and practical conservation actions that can be taken to help small ectotherms persist in modified human landscapes and cope with increasing temperatures due to climate change.

## Graphical abstract

Vegetation loss leads to habitat degradation, causing a decline in reptile species abundance due to loss of refuge, foraging opportunities, and exposure to high temperatures. Texas horned lizards have disappeared from many areas in Texas, but still occur in small towns with suitable habitat. Maintaining thermal refugia, such as shrubs and vegetation, is essential for the conservation of horned lizards and other small ectotherms in human‐modified landscapes facing increasing temperatures due to climate change.

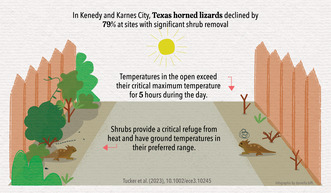

## 1 INTRODUCTION

Vegetation loss is a primary cause of habitat degradation and declines in reptile species abundance (Attum & Eason, 2006; Fleischner, 1994; Smith et al., 1996). This loss results in reduced prey diversity and number, increased predation risk due to loss of cover, and a reduction in important microhabitats required for thermoregulation (Jones, 1981; Norbury, 2001). Loss of vegetation leads to reptiles having to travel further to find prey, making them more vulnerable to predation (Hinsley, 2000; MacArthur & Pianka, 1966). Vegetation loss also reduces the availability of microhabitats for thermoregulation, hindering the ability of lizards to escape lethal substrate temperatures which is critical for species to persist in harsh and arid habitats (Adolph, 1990; Attum et al., 2013; Carrascal et al., 1992). When lizards are exposed to temperatures greater than their preferred body temperature, their activity is restricted, making them vulnerable to extinction due to climate change (Sinervo et al., 2010). The lower foraging efficiency caused by higher temperatures decreases the number and quality of offspring produced, leading to declining populations and eventual extinction (Sinervo et al., 2010).

Microhabitats that offer temperatures suitable for lizards determine how far they must move and how much energy they expend in finding an ideal thermal environment (Grbac & Bauwens, 2001; Sears et al., 2016). Reptile activity patterns are constrained by the distribution of microhabitats across space and time (Grbac & Bauwens, 2001). Heterogeneous landscapes support microhabitats with thermoregulatory patches that are variable in temperature and spatially closer together (Sears et al., 2016). This microhabitat configuration allows lizards to expend less energy moving to a favorable thermal patch to regulate body temperature and allows more time for foraging and reproductive opportunities (Sears et al., 2016). Homogeneous landscapes decrease available microhabitats and increase the distance lizards must travel between sun and shade, increasing their exposure to predators. Therefore, an understanding of the thermal regimes in different microhabitats is important to understand thermoregulatory behavior, habitat quality, and cost of living in diverse types of environments.

Lizards living in urban areas face additional challenges with thermal environments. Urban areas are often warmer and warm faster than natural areas due to concrete surfaces lowering albedo rates, thus increasing surface temperatures (Ackley et al., 2015; Kolbe et al., 2016; Taha, 1997). Research has shown that different types of landscaping in urban areas can significantly affect whether temperatures are within preferred temperature ranges for lizards (Ackley et al., 2015). Landscaping style (e.g., types of vegetation planted, fencing and borders, extent of tree canopy cover) can result in maximum daily air temperature differences up to 10°C between two adjacent habitats (Robinson et al., 2013; Todd & Andrews, 2008) and reduce surface temperatures over 10°C during the day (Brazel et al., 2007). Landscaping can also create habitat that can increase the diversity and abundance of reptiles in human modified areas (Ackley et al., 2015; Nopper et al., 2017; Pulsford et al., 2017). Few studies, however, have evaluated the thermal quality of microhabitats reptiles use in urban areas or how this might be related to abundance.

Texas horned lizards (*Phrynosoma cornutum*) need a mosaic of bare ground, sparse grass and herbaceous plants, and woody vegetation for unimpeded movement, foraging for ants, and thermoregulation (Anderson et al., 2017; Burrow et al., 2001; Eifler et al., 2012; Fair & Henke, 1998). Horned lizards are often active over longer periods of time than sympatric lizard species and display variable body temperatures which is attributed to relaxed thermoregulation (Pianka & Parker, 1975). Texas horned lizards have a high preferred body temperature (*T*_set_), ranging from 34.2 to 38.5°C and a high critical maximum temperature (CT_max_) (45.9–48.1°C) (Ballinger & Schrank, 1970; Kour & Hutchison, 1970; Lara‐Reséndiz, Gadsden, et al., 2015; Prieto Jr & Whitford, 1971; Russell, 2001; Table 1). These temperature tolerances are higher and more variable than other sympatric species of desert lizards (Pianka & Parker, 1975). Due to their ecology of being an ant specialist, it is likely that relaxed thermoregulation allows horned lizards to withstand direct sunlight for longer periods of time while foraging for ants in the open. Their cryptic camouflage and ability to withstand higher temperatures for longer aids in reducing predation risk since they do not have to move as frequently between sun and shade (Guyer & Linder, 1985; Pianka & Parker, 1975).

**TABLE 1:** Overview of preferred body temperatures (*T*_set_) of Texas horned lizards and *T*_set_ range (*T*_set25_ – *T*_set75_) in °C. Mean ± SE (standard error) or SD (standard deviation) reported for *T*_set_. ND is standard error that was not presented.

*N*	*T*_set_	*T*_set25_	*T*_set75_	Location	References
10	38.5 ± ND	37.5	39	Dona Ana Co., New Mexico	Prieto Jr and Whitford (1971)
97	34.2 ± 0.1 SE	32.5	36	Janos, Chihuahua, Mexico	Lemos‐Espinal and Smith (2009), Lara‐Reséndiz, Arenas‐Moreno, et al. (2015)
9	36.3 ± 2.39 SD	34.9	38.1	Dimmit/La Salle Co., Texas	Russell (2001)
19	35.7 ± 0.33 SE	33.5	38.5	Karnes Co., Texas	Present study

The Texas horned lizard is a threatened species in the state of Texas due to widespread population declines and a virtual disappearance in the eastern part of the state (Donaldson et al., 1994). These declines are attributed to habitat loss due to urbanization and agriculture, introduction of the red imported fire ant (*Solenopsis invicta*), loss of their preferred prey harvester ants (*Pogonomyrmex* spp.), and over‐collecting for the pet trade (Dixon, 2000; Donaldson et al., 1994; Henke, 2003). The species has remained a wildlife component of some small towns in Texas in areas that have suitable habitat, including Kenedy and Karnes City in south Texas that we have monitored since 2013. Lizards can occur at higher densities at some sites in town (52 lizards/hectare; Ackel, 2016) than are observed in more natural areas (3–10 lizards/hectare; Whitford & Bryant, 1979; Whiting et al., 1993). Our research has shown that the high density of lizards observed in these towns may be due to a variety of factors including isolation due to roads and buildings (which could increase horned lizard densities due to limited dispersal; Wall, 2014), a dietary shift to consuming smaller more abundant prey items (Alenius, 2018), and reduced predation pressure compared to natural areas by some types of predators (Mirkin et al., 2021).

We have noticed during our studies that horned lizards disappear or decrease in density at sites that have had shrubs and brush piles removed. The removal of shrubs could result in decline if it increases predation or degrades the thermal landscape (Gaudenti et al., 2021). Because predation pressure is lower in these towns than in more natural areas (Mirkin et al., 2021), we hypothesized that the primary cause of these declines is due to the loss of thermal refugia. In this study we first ask if horned lizard densities declined significantly at a site after shrub and brush pile removal using data we collected between 2013 and 2019. In 2019–2021, we determined body temperatures (*T*_*b*_) of horned lizards in the field and placed models with temperature loggers in three different microhabitats they utilize to determine if shrubs provide a better thermal environment than open areas or being buried under the soil in the open. In 2021, we determined the preferred body temperature (*T*_set_) in a laboratory gradient to better understand temperature preferences at our field sites. We used these data to ask if the thermal quality (*d*_*e*_) at a site is correlated with average lizard density. We also evaluated the accuracy (*d*_*b*_) and effectiveness (*d*_*e*_ − *d*_*b*_ and *E*) of thermoregulation of horned lizards in an urban environment.

## 2 METHODS

### 2.1 Study sites and fieldwork

**FIGURE 1:**
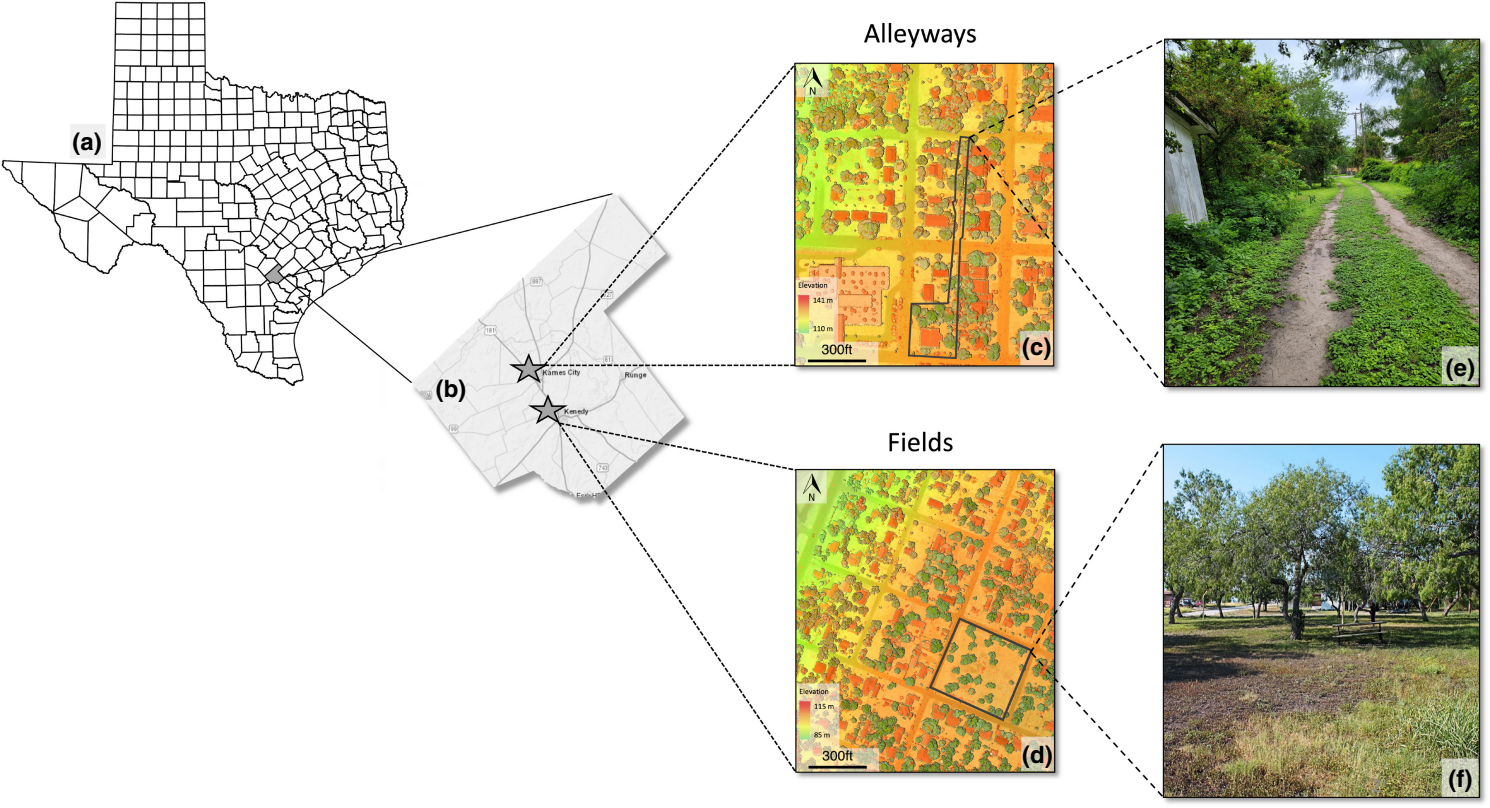
(a) Map of Texas counties, with Karnes County shown in gray. (b) Map of Karnes County, showing the locations of Kenedy and Karnes City. (c and d) Aerial map showing classification of trees and shrubs (dark green), houses (red), and ground (color ramp representing range of elevation in meters) from two of our study sites (outlined in black) within Karnes City and Kenedy, respectively. Maps generated by classifying LiDAR point cloud data (United States Geological Survey, TX Hurricane B4 2018, Date: 2019‐01‐12Z–2019‐02‐21Z, Quality: QL 2) using Esri ArcGIS® Pro. Study sites are split up into two types of spatial structure: (c) alleyways, which are more heterogeneous in structure and thermal microhabitats, and (e) have dirt roads bordered by houses, fences, and vegetation (i.e., tree canopy cover, ornamental shrubs, and native vegetation), and (d) fields, which are less heterogenous and have thermal microhabitats spread apart since fields have (f) short vegetation cover interspersed with clumps of trees and shrubs.

Texas horned lizards have been studied since 2013 in two small towns (~3042–3296 people) in south Texas; Kenedy (28.8191° N, 97.8486° W; elevation = 81 m) and Karnes City (28.8850° N, 97.9008° W; elevation = 131 m). Between 2013 and 2021, we surveyed 16 sites (3 in Kenedy and 13 in Karnes City) (Table 2). Each site was surveyed between 4 and 9 years (average 8 years). The sites are in school yards, alleyways, and abandoned lots that vary in size from 0.07 to 1.11 hectares (Alenius, 2018). The habitat at these sites is dominated by honey mesquite (*Prosopis glandulosa*), anacua (*Ehretia anacua*), and sugarberry (*Celtis laevigata*) along with native grasses, forbs, and ornamental plants. These sites are classified into two groups based on spatial structure: alleyways and fields connected to alleyways, which have a dirt road bordered by houses, fences, and vegetation (i.e., tree canopy cover, ornamental shrubs, and native vegetation) and fields, which have short vegetative cover interspersed with clumps of trees and shrubs (Figure 1).

**TABLE 2:** Average ± SE Texas horned lizard density at 16 surveyed sites in Karnes City (KC) and Kenedy (Ken), Texas, USA. *N* years is the number of years a site was surveyed. Year of major vegetation removal at a site and lizard density before and after major vegetation removal are presented (see text). *T*_*e*_ models is the number of temperature loggers placed at each site.

Site	Habitat type	Town	HL/ha	*N* years	Year veg. Removed	HL/ha year before veg. removal	HL/ha year after veg. removal	*T*_*e*_ models
1	Field	KC	8.12 ± 2.74	8	2016	13.92	0.00	3
2	Alley	KC	70.24 ± 12.00	9	NA	NA	NA	3
3	Field	KC	14.41 ± 2.32	5	NA	NA	NA	0
4	Field	KC	6.57 ± 1.39	9	2018	6.76	1.69	0
5	Field/Alley	KC	37.65 ± 6.91	8	NA	NA	NA	3
6	Alley	KC	26.46 ± 17.55	7	2016	51.09	0.00	0
7	Alley	KC	51.06 ± 20.64	5	2019	117.02	31.91	3
8	Field/Alley	KC	93.9 ± 28.41	9	2017	158.25	50.51	3
9	Field	KC	19.63 ± 5.33	9	2019	20.08	8.03	6
10	Field	KC	1.23 ± 0.82	8	2016	6.56	2.46	0
11	Field	KC	2.51 ± 1.95	8	2017	25.42	0.00	3
12	Alley	KC	39.56 ± 8.12	7	NA	NA	NA	0
13	Alley	KC	66.78 ± 12.41	9	2018	106.38	69.15	3
14	Field	Ken	3.24 ± 1.28	9	2016	7.30	0.00	3
15	Field	Ken	13.65 ± 6.22	9	2017	35.09	8.77	3
16	Field	Ken	3.67 ± 1.64	9	2017	2.92	0.00	6

Sites are surveyed by walking transects with 2–4 people between 8:00–12:00 and 17:00–20:00, which corresponds to active periods for Texas horned lizards (Ackel, 2016; Moeller et al., 2005). One of the study's authors (Williams) was present at all transects during this study. Transects are conducted at each site 8 times each summer, divided up into three sampling periods: 2 weeks in late May/early June (each site is surveyed 4 times), 1 week in early July (each site is surveyed 2 times), and 1 week in late July and early August (each site is surveyed 2 times). Upon capture, we record time, sex, weight (g), length (mm), and location using ArcGIS Collector.® We photograph belly spots for individual identification.

We noted when the sites experienced major changes to their vegetation, such as removal of shrubs along fence rows, removal of isolated bushes and bushes around the bases of trees in parks, and removal of large brush piles. Mowing and trimming of grasses and forbs, which usually occurred several times during the summer months at all sites, was not counted as major vegetation removal. We compared horned lizard density (lizards/hectare) in the year before vegetation removal and the year after vegetation removal using a Wilcoxon signed‐rank test.

Average annual temperature for both towns is 18°C. Air temperature is higher in the summer, reaching an average of 36°C and a maximum temperature of 40°C in the shade. Average annual precipitation is 790 mm and monthly averages vary from 33 to 76.2 mm, with May receiving the most rainfall and December receiving the least. Rainfall patterns are typically higher during the beginning of the field season in late May and continue to decrease through August.

### 2.2 Body temperature in the field (*T*_*b*_)

In 2019–2021, field cloacal temperature (*T*_*b*_) was recorded within 30 s of capture by inserting a small temperature probe connected to a digital thermometer (GDEALER Model DT8; accuracy ±1°C; resolution ±0.1°C) one centimeter into the cloaca. During the 2020–2021 field seasons, the microhabitat where the lizard was found was also recorded and classified as open sunny, open overcast, or shade/vegetation.

### 2.3 Operative environmental temperatures (*T*_*e*_) and model calibration

**FIGURE 2:**
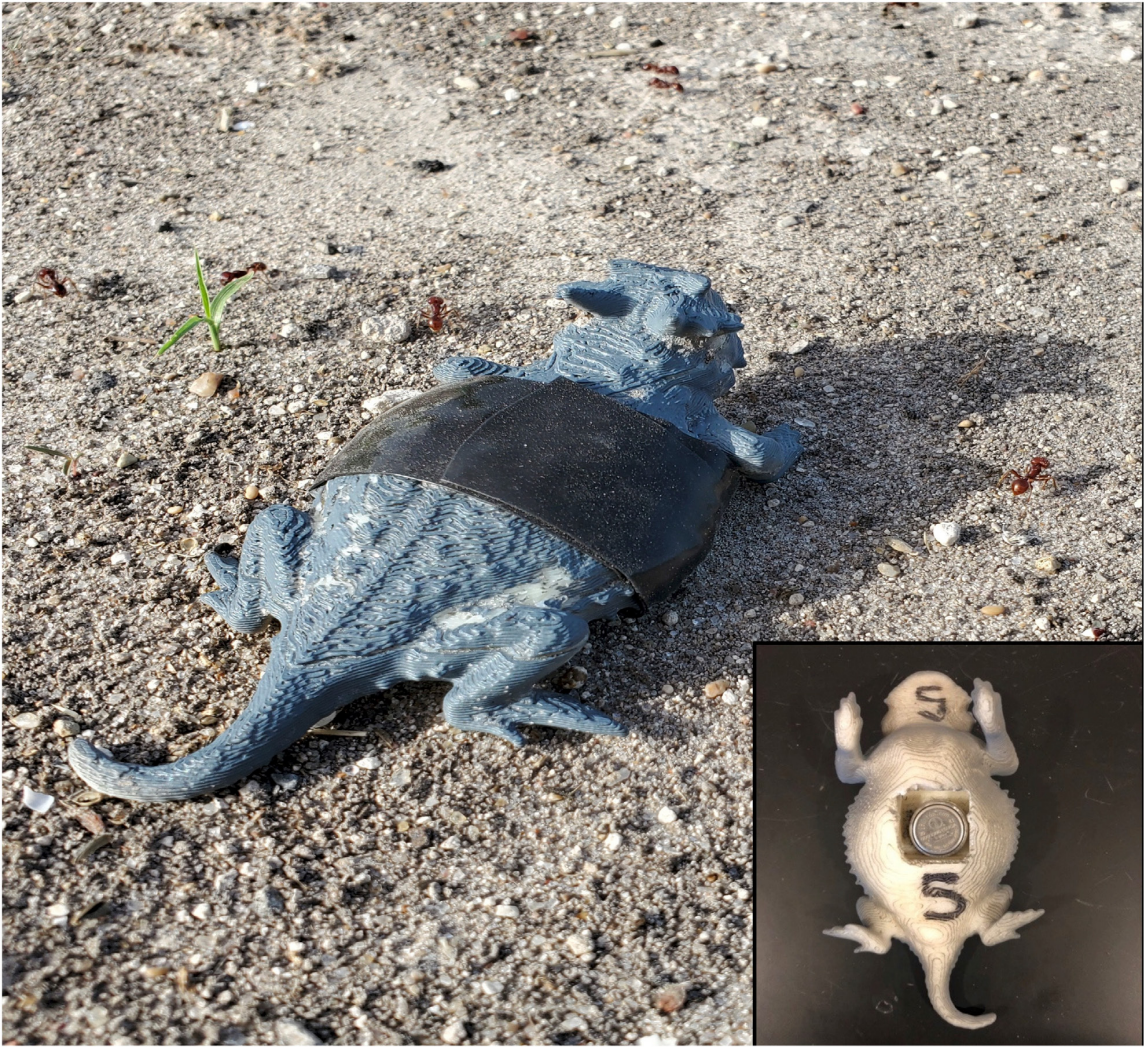
Horned lizard model equipped with a temperature logger embedded in the belly area and secured with black self‐fusing repair tape to approximate environmental temperatures (*Te*) available to lizards.

In 2019–2021, we determined environmental temperatures (*T*_*e*_) at sites that currently have horned lizards and sites where they have disappeared. *T*_*e*_ has historically been determined using copper or polyvinylchloride (PVC) models to estimate available temperatures for small ectotherms, but we used 3D printed models of adult Texas horned lizards for morphological accuracy (Mirkin et al., 2021; Watson & Francis, 2015). Models were printed with acrylonitrile butadiene styrene (ABS) and painted with 33% reflective paint (Rustoleum™ gray primer) that corresponds to the reflectivity of horned lizards (Adolph, 1990; Lara‐Reséndiz, Gadsden, et al., 2015). The underside of the model had a recessed opening that held a DS1922L Thermochron™ temperature logger that records temperature at a resolution of ±0.2°C (Figure 2). Self‐fusing repair tape was used to seal the temperature logger in the model. The addition of this tape did not significantly change the temperatures recorded by the temperature loggers (models with and without tape, *y* = 0.97*x*, *n* = 29, *R* ^2^ = .999).

In 2019, 15 models were placed at 5 sites from June 2 – 8th. After we could purchase more temperature loggers, 30 models were placed at 10 sites from June 30 – July 6th and August 1 – 8th. In 2020 and 2021, 45 models were placed at 13 sites from June 1– June 14th, July 1 – 6th, and August 1 – 6th. Of these 13 sites, 11 were sites that were regularly surveyed and two of the sites were fields in Kenedy where lizards historically occurred but disappeared in the late 1990s (Wade Phelps personal communication; Table 2). We checked those two sites several times every year since 2013 for horned lizards and scat but never found evidence they had recolonized those areas. At each site, one model was placed in the open, one under vegetation (shrubs at 12 sites and thick grass under tree canopies at 3 sites), and one buried ~2 cm under the soil surface in open areas to mimic the three common microhabitats Texas horned lizards utilize (Burrow et al., 2001; Wall, 2014). The two largest sites had two sets of three models (*n* = 6) placed as far apart as possible to provide better coverage (Table 2). Models were placed in areas where horned lizards had been previously observed for all the sites by using prior GPS locations from ArcGIS Collector.® Models were placed in the same sites and the same microhabitat locations each year. Temperature loggers were programmed to record temperature every 10 min from 7:00 to 20:00 to estimate environmental temperatures (*T*_*e*_) throughout the horned lizard's activity period (Lara‐Reséndiz, Gadsden, et al., 2015). We calculated mean *T*_*e*_ values for each time point (every 10 min during the lizard's active period) for each month and year for open, buried, and vegetation microhabitats. The models were calibrated against live lizards by first capturing a lizard and measuring the cloacal temperature (*T*_*b*_) using a small temperature probe connected to a digital thermometer (GDEALER Model DT8; accuracy ±1°C; resolution ±0.1°C) within 30 s of capture and then placing a model in the same spot as the captured lizard and recording the temperature on the logger after 10 min (Dzialowski, 2005; Kolbe et al., 2016). Hourly ambient air temperatures were determined by using publicly available historic weather data from the KBEA weather station in Beeville, TX (28.4008° N, 97.7483° W; altitude = 64 m), which is located approximately 50 km away.

### 2.4 Preferred body temperature in the laboratory (*T*_set_)

In 2021, *T*_set_ was recorded using a laboratory thermal gradient that consisted of a plastic box 88.6 cm × 42.2 cm × 15.6 cm (length, width, and height) that was filled with 2–3 cm of sand (Angilletta, 2009; Hertz et al., 1993; Sinervo et al., 2010). At one end of the box, a Phillips™ 250 W heat light bulb was placed 33.7 cm above the substrate to create a thermal gradient ranging from 25 to 60°C. The gradient was housed in a climate‐controlled room at a constant temperature of 20°C. We captured adult lizards (>69 mm, *n* = 19) in the field and allowed them to acclimate in the thermal gradient overnight (between 12 and 23 h).

Lizards were exposed to natural lighting through windows and artificial lighting, which mimicked the natural daylight cycles at our field location. No food or water was offered during the experiments given the short captive period and their natural history (i.e., ant specialist and do not drink frequently), but enclosures were kept humid with a damp towel during the acclimation period. Lizards were placed in the middle of the gradient when the trial started. Preferred body temperature (*T*_set_) was measured every 2 h from 8:00 to 20:00 with the same temperature probe used for *T*_*b*_. The thermal gradient was divided into four quadrants (in order from closest to heat source to furthest: front, first quarter, second quarter, and back) and temperature was recorded every 2 h using a Etekcity Lasergrip 774 infrared thermometer (resolution ±0.1°C) held 3 cm above the substrate. After the experiment, all lizards were released at their capture site. The 25 and 75 percent quartiles for preferred body temperature (*T*_set25_ – *T*_set75_) were used as the upper and lower *T*_set_ (Hertz et al., 1993; Lara‐Reséndiz, Gadsden, et al., 2015).

### 2.5 Habitat thermal quality and thermoregulatory indices

Following methodology from Hertz et al. (1993), data from *T*_*b*_, *T*_set_, and *T*_*e*_ were used to calculate the accuracy of thermoregulation (*d*_*b*_) and habitat thermal quality (*d*_*e*_) as follows: if *T*_*b*_ or *T*_*e*_ < *T*_set_, then *d*_*b*_ = *T*_*b*_ − *T*_set25_ and *d*_*e*_ = *T*_*e*_ − *T*_set25_, and if *T*_*b*_ or *T*_*e*_ > *T*_set_ then *d*_*b*_ = *T*_*b*_ − *T*_set75_ and *d*_*e*_ = *T*_*e*_ − *T*_set75_, respectively. When *T*_*b*_ or *T*_*e*_ values were within *T*_set_ range, *d*_*b*_ and *d*_*e*_ were considered equal to zero. High values of *d*_*b*_ and *d*_*e*_ indicate low accuracy and low thermal quality, while values equal to or near zero indicate high accuracy of thermoregulation and represent thermally ideal environments. Thermoregulatory effectiveness (*E*) was then calculated using ${\bar{d}}_{b}$ and ${\bar{d}}_{e}$, where the overbars represent mean values of the deviations, using the following equation: *E* = 1 − (${\bar{d}}_{b}/{\bar{d}}_{e}$). We calculated ${\bar{d}}_{e}$ using *T*_*e*_ temperatures from 8:00–12:00 and 17:00–20:00 since field cloacal *T*_*b*_ temperatures (and therefore ${\bar{d}}_{b}$) were only measured during those time periods. When calculating *E*, we used the mean value of ${\bar{d}}_{e}$ for all microhabitats across each time point. Given that *d*_*e*_ did not vary between years (One‐way ANOVA, *F*_2,223_ = 2.2, *p* = .11), we averaged *d*_*e*_ across years to obtain ${\bar{d}}_{e}$. An *E* value near to one indicates an organism that actively thermoregulates because environmental temperature is far from its preferred temperature. These lizards are under thermal stress and must increase or decrease their *T*_*b*_ with respect to *T*_*e*_. An *E* value equal or near to zero indicates a thermoconformer, which is not regulating temperatures actively since the environmental temperature is within its preferred temperature range (Hertz et al., 1993). However, an *E* value can come from a variety of combinations of ${\bar{d}}_{b}$ and ${\bar{d}}_{e}$ (Hertz et al., 1993). For example, a species could occupy a difficult thermal environment (i.e., high *d*_*e*_ values) and utilize a different thermoregulatory strategy compared to a species that occupies a more benign thermal environment, but these species could still have the same *E* value if the ratios between ${\bar{d}}_{b}$ and ${\bar{d}}_{e}$ are the same (Blouin‐Demers & Weatherhead, 2001). Thus, it is also important to consider the difference in magnitude between ${\bar{d}}_{b}$ and ${\bar{d}}_{e}$ when interpreting *E* (Blouin‐Demers & Nadeau, 2005). Another way to calculate thermoregulatory effectiveness is by using the following equation: ${\bar{d}}_{e}–{\bar{d}}_{b}$. This method avoids the limitations associated with ratios and can quantify the extent of departure from perfect thermoconformity with values of zero representing thermoconformity and positive values indicating thermoregulation (Blouin‐Demers & Weatherhead, 2001). We calculated ${\bar{d}}_{e}–{\bar{d}}_{b}$ using *T*_*e*_ (and therefore *d*_*e*_) data from 8:00–12:00 and 17:00–20:00 as we did for *E*.

When calculating *d*_e_ for each site (*n* = 13), we used *T*_*e*_ temperatures from 8:00–20:00 across all microhabitats for each month and year for that site. We calculated *d*_e_ for each microhabitat (open, buried in dirt, and underneath vegetation) for all months and years to measure the average thermal quality of the microhabitats available to horned lizards during their active period. We also calculated the percent time each microhabitat *T*_*e*_ (open, buried in dirt, or under vegetation) fell within their preferred temperature range (*T*_set25_ – *T*_set75_) and exceeded their critical thermal maximum (CT_max_). We then used Kruskal–Wallis and Dunn's post hoc tests to examine differences between microhabitats. Hours of restriction (*h*_*r*_) were expressed as the hours in each day that *T*_*e*_ exceeds CT_max_ (Ivey et al., 2020; Taylor et al., 2021).

### 2.6 Statistical analysis

#### 2.6.1 Body temperature in the field (*T*_*b*_) and model calibration

We performed generalized linear models (Minitab® Version 19) to explore the variability in field cloacal temperatures (*T*_*b*_) among lizards (Appendix: Tables A1 and A2). Recaptured lizards within the same year were identified by belly spots and only the first *T*_*b*_ measurement was included in analysis to avoid pseudoreplication. *T*_*b*_ temperatures from 2019 to 2021 were added as the response variable; time of capture (grouped into 2‐h blocks), month, year, age, and sex were added as factors; and body condition (weight/SVL) was added as a covariate (Appendix: Table A1). Our second model excludes data from 2019 because microhabitat information was not recorded during that year (Appendix: Table A2). This model includes all previous terms and adds microhabitat classification (open sunny, open overcast, shade/vegetation) as a factor (Appendix: Table A2). We started with the full model with all relevant interaction terms and proceeded with stepwise selection. The model with the lowest bias‐corrected Akaike information criterion (AIC_c_) score was selected. Tukey post hoc tests were then performed on significant factors to see what groups were different from one another. Microsoft Excel® and Minitab® Version 19 were used for regression analysis to examine calibration between cloacal body temperature (*T*_*b*_) with environmental operative temperatures measured by models (*T*_*e*_). We used the student's *t* distribution (*t* = (slope − 1)/SE with df = *n* − 2) to test if the slope was significantly different than one.

#### 2.6.2 Preferred body temperature (*T*_set_)

We performed a mixed effects model (Minitab® Version 19) with lizard ID as a random effect, month and time(month) as fixed effects, and gradient quadrant temperatures (in order from closest to heat source to furthest: front, first quarter, second quarter, and back) as covariates (Appendix: Table A3). We used a mixed effects model to account for repeated measurements of the same lizard in the thermal gradient. We then found the model predicted means and compared them to the observed means to find our mean preferred body temperature (Camacho & Rusch, 2017). We performed a student's *t*‐test to see if there was a significant difference between model predicted means and the observed mean preferred temperature.

#### 2.6.3 Habitat thermal quality (*d*_*e*_)

Assumptions of normality were analyzed visually and by using Kolmogorov–Smirnov test. Homogeneity of variances were analyzed using Levene's test. To explore differences in thermal quality (*d*_*e*_) between years and microhabitats, we used one‐way ANOVA and Tukey post hoc test and Kruskal–Wallis and Dunn's post hoc test, respectively. Student's *t*‐test was used to explore differences in *d*_*e*_ between alleyways and fields. Microsoft Excel® and Minitab® Version 19 were used for spearman rank correlation to see if average lizard density (lizards/hectare) correlated to site thermal quality (*d*_*e*_). Statistical significance is set at α=0.05. Mean ± standard error is presented in the results, including figures and tables unless otherwise stated.

## 3 RESULTS

### 3.1 Change in density

Horned lizard density averaged 28.67 ± 7.15 individuals at our sites (*n* = 16 sites, range 1.23–93.9; Table 2). Horned lizard density was higher in alleys (55.09 ± 8.80, *n* = 7) than in fields (8.11 ± 2.13, *n* = 9) (*t*‐test unequal variances, *t*_7_ = 5.19, *p* = .001). There were only four sites which did not experience major vegetation removal between 2013 and 2021, and horned lizards declined an average of −0.04 ± 0.08% at these four sites. Twelve sites experienced major vegetation and brush removal between 2013 and 2021 (Table 2). At four of these sites, vegetation that was removed grew back, and lizards recolonized one of these sites. At eight sites, the vegetation removed did not grow back and all lizards disappeared at five of those sites. The density of lizards at a site decreased by 79% in the year after vegetation removal (median density before removal = 22.75; median density after removal = 2.08, *W* = 78, *n* = 12, *p* = .002; Table 2).

### 3.2 Body temperature in the field (*T*_*b*_)

**FIGURE 3:**
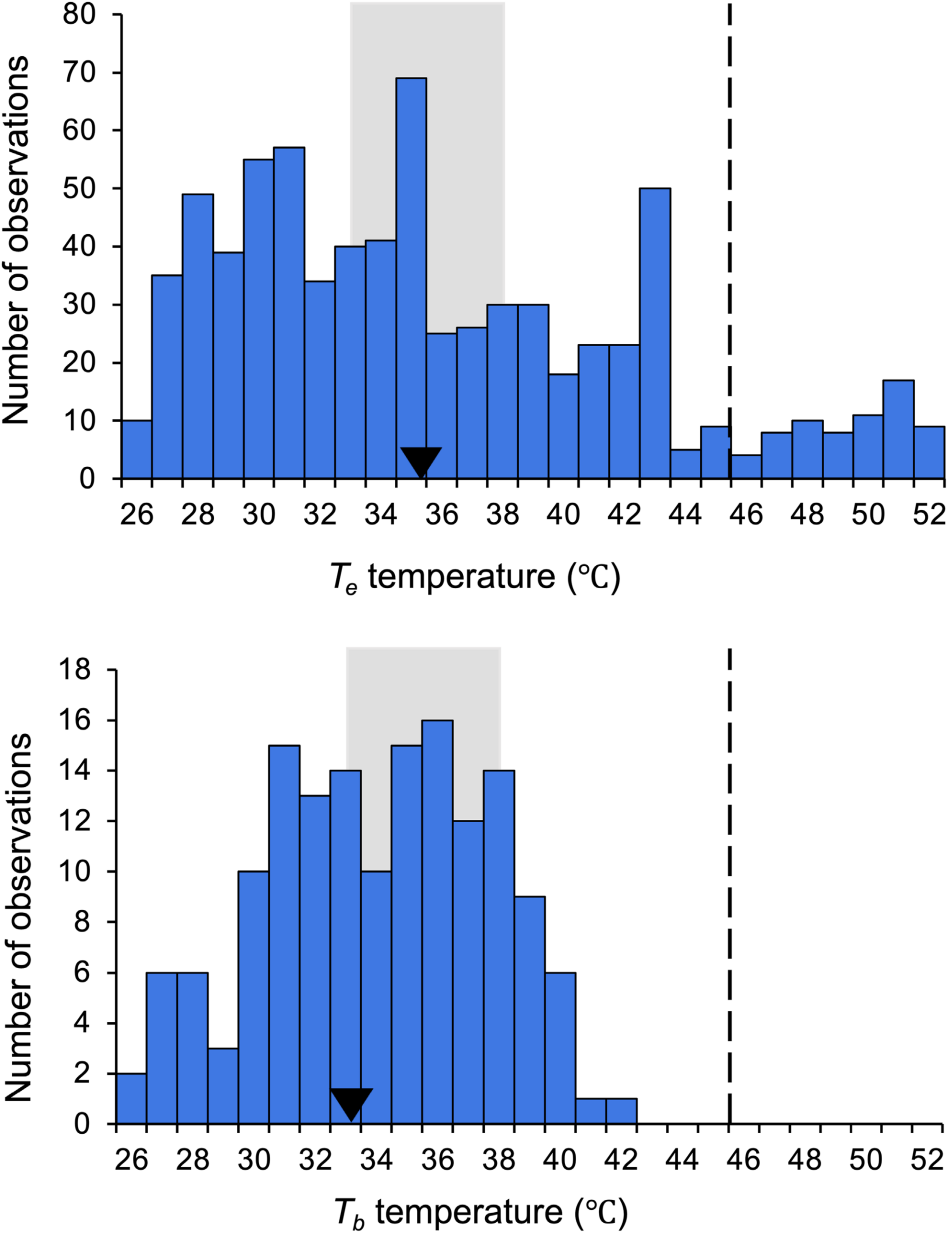
Frequency distribution of environmental temperatures *T*_*e*_ (top) and horned lizard *T*_*b*_ cloacal temperatures (bottom) from Karnes Co., Texas, 2019–2021. Mean ± SE *T*_*e*_ was 35.2 ± 1.1°C (*n* = 326 models) and mean *T*_*b*_ was 33.6 ± 0.3°C (*n* = 153 lizards). Black arrows represent the mean, the gray box represents their preferred temperature (*T*_set_) interquartile range (33.5–38.5°C), and the black dashed line represents their critical thermal maximum (CTmax = 45.9°C; Prieto Jr & Whitford, 1971).

One hundred and fifty‐three *P. cornutum* were captured (66 in 2019, 47 in 2020, and 40 in 2021) and their body temperature (*T*_*b*_) recorded. Of the 102 individuals for which we had microhabitat data, 37 were in the open under sunny conditions, 17 were in the open under overcast conditions, and 48 were in the shade of vegetation. The overall mean *T*_*b*_ was 33.6 ± 0.30°C (*n* = 153, range = 23.6–41.2°C; Figure 3). The distribution of *T*_*b*_ and *T*_*e*_ indicates that horned lizards avoided higher temperatures and used microhabitats that kept their mean *T*_*b*_ slightly lower than mean environmental temperatures (Figure 3).

Body temperature (*T*_*b*_) was different between years (*F*_2,141_ = 5.66, *p* = .004) and was significantly lower in 2021 (*n* = 40, 32.3 ± 0.60°C) than in 2019 (*n* = 57, 33.7 ± 0.48°C; Tukey, *p* = .034), and 2020 (*n* = 46, 34.2 ± 0.53°C; Tukey, *p* = .002). Body temperature (*T*_*b*_) fluctuated through time of day (*F*_3,140_ = 18.54, *p* < .00001) with early morning temperatures (“8:00 & 9:00” category) being significantly cooler than all later time categories (“10:00 & 11:00” difference in means = −3.10 ± 0.64°C, Tukey, *p* < .0001; “17:00 & 18:00” difference in means = −5.10 ± 0.76°C, Tukey, *p* < .001; “19:00 & 20:00” difference in means = −3.67 ± 0.98°C, Tukey, *p* = .002). There was no significant difference in *T*_*b*_ between months (*F*_2,141_ = 0.21, *p* = .81), age (*F*_1,142_ = 1.03, *p* = .31), sex (*F*_1,142_ = 0.02, *p* = .88), or body condition (*F*_1,142_ = 0.32, *p* = .57). Body temperature (*T*_*b*_) during 2020–2021 differed by microhabitat (*F*_2,83_ = 7.64, *p* = .001) with lizards found in the shade of vegetation being on average 3 ± 0.70°C cooler than ones found in open sunny microhabitat conditions (Tukey, *p* < .001).

### 3.3 Preferred body temperature in the laboratory (*T*_set_)

Individual as a random variable did not explain variability in *T*_set_ temperatures (*p* = .07). Time of day nested in month (*F*_15,80_ = 0.98, *p* = .49) and month (*F*_2,16_ = 1.99, *p* = .17) also had no significant effect on *T*_set_. Model predicted means averaged to 36 ± 0.47°C. We decided to use the observed mean of 35.7 ± 0.33°C since it was not statistically different than the model predicted mean (*t*_0.05(2),26_ = 0.45, *p* = .65). Therefore, the preferred body temperature in the thermal gradient was 35.7 ± 0.33°C (*n* = 19, range = 27.2–41.5°C). The *T*_set_ interquartile range (*T*_set25_ – *T*_set75_) was 33.5–38.5°C.

### 3.4 Operative environmental temperatures (*T*_*e*_)

There was a highly significant linear relationship between field *T*_*b*_ and model estimated *T*_*e*_ (*y* = 0.80*x* + 6.57, *R* ^2^ = .89, *p* = .02) and the slope was not significantly different than 1.0 (*n* = 71, *t*_0.05(2),69_ = −0.159, *p* = .87), suggesting that models accurately measured *T*_*e*_ available to horned lizards during their active hours.

**FIGURE 4:**
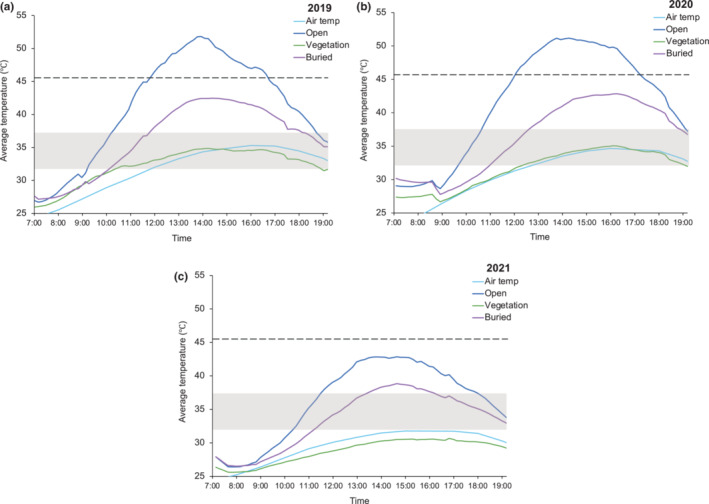
Average operative temperatures (*T*_*e*_) over Texas horned lizard's active period measured by models in open, buried in the open, and vegetation microhabitats for (a) 2019, (b) 2020, and (c) 2021. The dark blue line represents open, purple line represents buried in the open, green line represents vegetation, and the light blue line represents hourly ambient air temperatures from Karnes Co., Texas. The gray box represents their preferred temperature (*T*_set_) interquartile range (33.5–38.5°C). The black dashed line represents their critical thermal maximum (CTmax = 45.9°C; Prieto Jr & Whitford, 1971).

Environmental temperatures (*T*_*e*_) for open microhabitats averaged to 40.8 ± 0.83°C in 2019 (*n* = 88, range = 26.7–51.8°C); 41.3 ± 0.91°C in 2020 (*n* = 79, range = 28.7–51.2°C); and 36.1 ± 0.64°C in 2021 (*n* = 78, range = 26.4–42.9°C). Average open temperatures exceeded the critical maximum temperature (CT_max_) for 5 h in the middle of the day in 2019 and 2020 and were considered hours of restricted activity (*h*_*r*_). Open temperatures never reached CT_max_ in 2021 but exceeded the upper preferred temperature (*T*_set75_) for 5 h during the middle of the day (Figure 4). Open microhabitats fall in preferred temperatures in the morning (9:00–10:00) and are important for increasing body temperature during the beginning of their activity period (Figure 4).

Environmental temperatures for buried in the open microhabitats averaged 36.1 ± 0.50°C in 2019 (*n* = 88, range = 27.2–42.5°C); 36.3 ± 0.58°C in 2020 (*n* = 79, range = 27.8–42.8°C); and 33.4 ± 0.47°C in 2021 (*n* = 78, range = 26.5–38.8°C). Buried microhabitat temperatures never reached CT_max_ in all 3 years, but temperatures exceeded the upper preferred temperature (*T*_set75_) for 6 h in the middle of the day in 2019 and 2020, which would require lizards to seek refuge elsewhere to stay within their preferred temperature range (Figure 4). Buried microhabitat temperatures stayed within the *T*_set_ range for most of the day in 2021 (Figure 4).

Environmental temperatures for vegetation microhabitats averaged 32.1 ± 0.28°C in 2019 (*n* = 88, range = 26–34.9°C); 31.6 ± 0.32°C in 2020 (*n* = 79, range = 26.7–35.1°C); and 28.6 ± 0.29°C in 2021 (*n* = 78, range = 25.6–30.7°C). Vegetation microhabitats provided temperatures within the *T*_set_ range during the hottest parts of the day in 2019 and 2020, when open and buried microhabitats were above preferred temperatures or sometimes above CT_max_ (Figure 4). Vegetation temperatures never reached *T*_set_ in 2021 and stayed below their preferred temperature range the entire day (Figure 4). Ambient air temperatures were closest to temperatures found under vegetation, which is expected since temperature data are measured in the shade (Figure 4).

There was a significant difference in percent time that *T*_*e*_ was at critical maximum temperature (*F*_2,323_ = 13.09, *p* < .0001) and percent time that *T*_*e*_ was at preferred temperatures (*F*_2,323_ = 2.95, *p* = .05) between years. This difference was due to 2021 being on average cooler than 2019 and 2020 (Tukey, *p* < .05 both cases). After looking at temperature abnormalities at our field sites, 2021 was the only year that temperatures were cooler on average since monitoring this population starting in 2013 (NOAA Climate at a Glance: Global Time Series). We therefore decided to remove 2021 *T*_*e*_ from the percent time at critical temperature and percent time at preferred temperature analyses below to give a more representative view of the temperatures commonly experienced by lizards at our sites.

**FIGURE 5:**
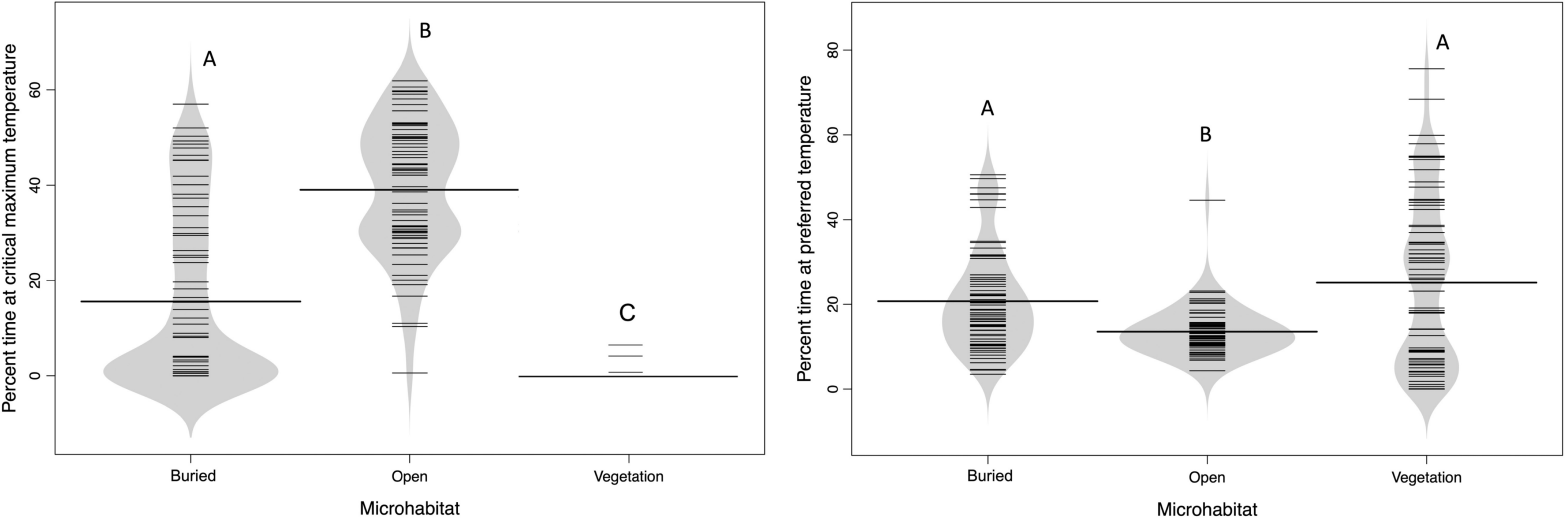
Bean plot showing percent time *T*_*e*_ was at critical temperature (CTmax) (left) and at preferred temperatures (*T*_set25_ – *T*_set75_) (right) for three different microhabitat classifications in Karnes Co., Texas from 2019 to 2020. Bold horizontal lines represent the average for each microhabitat, and smaller lines correspond to the average values of each model within that microhabitat. Groups sharing a letter are not significantly different from each other whereas different letters are significantly different from each other (buried in the open, *n* = 68 models; open, *n* = 63 models; and vegetation, *n* = 70 models).

Buried in the open microhabitat was above their critical temperature (CT_max_) for 15.6% of the day and within their preferred temperature range (*T*_set25_–*T*_set75_) for 20.7% of the day. Open microhabitat was above their critical temperature for 39.1% of the day and within their preferred temperature range 13.5% of the day. Vegetation microhabitat was above their critical temperature for 0.3% of the day and within their preferred temperature range 25.1% of the day (Figure 5). All microhabitats were significantly different from each other for percent time at critical temperature (Figure 5; Dunn, *p* < .001). Percent time at preferred temperature was only significantly different between buried and open and vegetation and open microhabitats (Figure 5; Dunn, *p* < .001).

### 3.5 Habitat thermal quality (*d*_*e*_)

**FIGURE 6:**
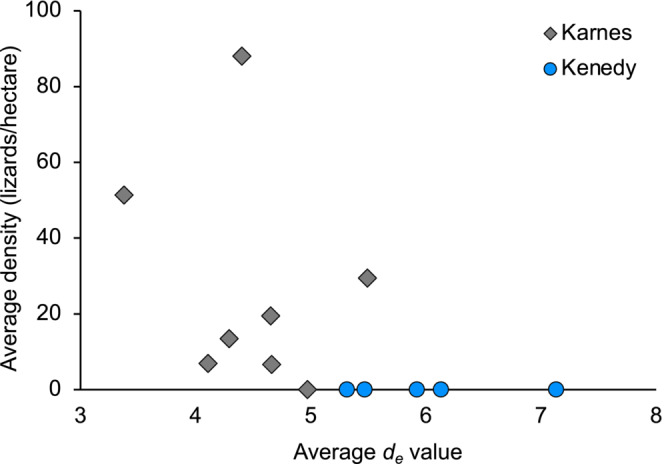
Scatterplot showing the relationship between average thermal quality score (*de*) and average density of horned lizards (lizards/hectare) for 2019–2021 in two small towns Kenedy (blue circle) and Karnes City (gray diamond), Texas (*n* = 13 sites).

**FIGURE 7:**
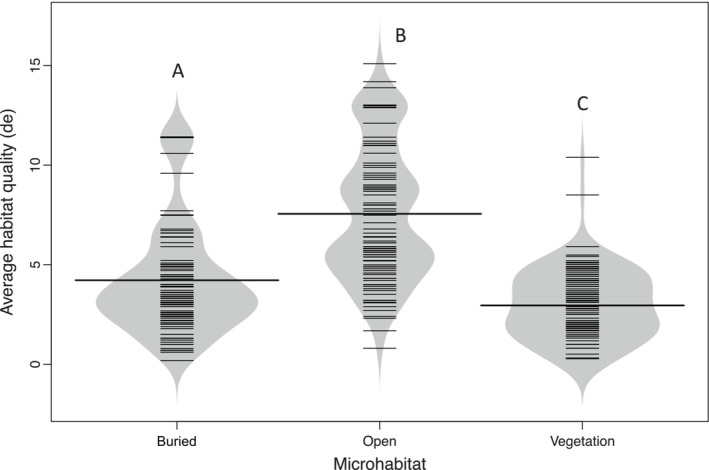
Bean plot showing the distribution of quality scores (*de*) for three different microhabitat classifications in Karnes Co., Texas from 2019 to 2021. Bold horizontal lines represent the average *d*_*e*_ for each microhabitat, and smaller lines correspond to the average *d*_*e*_ values of each model within that microhabitat. Groups sharing a letter are not significantly different from each other whereas different letters are significantly different from each other (buried, *n* = 105 models; open, *n* = 103 models; and vegetation, *n* = 118 models).

Habitat thermal quality (*d*_*e*_) did not differ between years (2019–2021; One‐way ANOVA, *F*_2,223_ = 2.2, *p* = .11) and therefore data were pooled and $\bar{d}$_*e*_ = 2.30 ± 0.19 (Table 3). There was a negative correlation between the average density of horned lizards (lizards/hectare) and average thermal quality (*d*_*e*_) across sites (*r*_*s*_ = −0.68, *p* = .01; Figure 6), meaning as thermal quality degraded (i.e., higher *d*_*e*_ values) horned lizard density decreased. Thermal quality was higher (i.e., lower *d*_*e*_ value) for Karnes City, which still has a good population of horned lizards (*d*_*e*_ = 4.5 ± 0.22), compared to Kenedy, which has experienced steep declines and had no horned lizards present in 2019–2021 (*d*_*e*_ = 6.0 ± 0.32; *t*_0.05(2),8_ = −3.85, *p* = .005). Average thermal quality (*d*_*e*_) was highest (i.e., lower value) for vegetation followed by buried then open microhabitats (Dunn, *p* < .001 in both cases; Figure 7). Thermal quality was higher for sites that contained alleyways (*d*_*e*_ = 4.4 ± 0.70) rather than fields (*d*_*e*_ = 5.5 ± 0.90; *t*_0.05(2),12_ = −2.78, *p* = .017).

**TABLE 3:** Field body temperature (*T*_*b*_), operative environmental temperature (*T*_*e*_), preferred temperature in laboratory (*T*_set_) and *T*_set_ range (*T*_set25_–*T*_set75_) in °C, and accuracy of thermoregulation (*d*_*b*_), habitat thermal quality (*d*_*e*_), and thermoregulatory effectiveness (*d*_*e*_ − *d*_*b*_ and *E*). Showing mean ± SE.

Species	*T*_*b*_	*T*_*e*_	*T*_set_	*T*_set_ range	*d*_*b*_	*d*_*e*_	*d*_*e*_ *− d*_*b*_	*E*
*Phrynosoma cornutum*	33.6 ± 0.3	35.2 ± 1.1	35.7 ± 0.33	33.5—38.5	1.59 ± 0.2	2.30 ± 0.2	0.71	0.31

### 3.6 Thermoregulatory indices

The average deviations of *T*_*b*_ from *T*_set_ range (i.e., ${\bar{d}}_{b}$) was low (1.59°C), indicating the lizards were active close to their preferred temperature range and suggesting that *P. cornutum* exhibits accurate thermoregulation (${\bar{d}}_{b}$ = 1.59; Table 3). Effectiveness of thermoregulation (*E*) was 0.31, indicating *P. cornutum* is a moderate thermoregulator. Effectiveness of thermoregulation for ${\bar{d}}_{e}–{\bar{d}}_{b}$ was 0.71, also indicating some thermoregulatory behavior and a thermally benign environment (Table 3).

## 4 DISCUSSION

In tropical and desert areas the major challenge for lizards is to lower their body temperature and vegetation plays a key role in providing shade and cooling temperatures (Grimm‐Seyfarth et al., 2017; Kearney et al., 2009). Urban areas located in these environments have additional challenges with increased surface temperatures, although landscaping can significantly affect whether temperatures are within preferred temperature ranges for lizards (Ackley et al., 2015). We found that shrubs in town provide a critical refuge for Texas horned lizards and represent the highest quality microhabitat (*d*_*e*_) that is closest to their preferred temperature range. Environmental temperatures (*T*_*e*_) underneath vegetation never exceeded the lizards' upper *T*_set75_ or CT_max_, whereas temperatures in the open often exceeded CT_max_ or were above their upper preferred temperature (*T*_set75_) for 5 h in the middle of the day during their active period. We found when shrubs and brush piles had been removed from fields, fence rows, and the base of trees, the number of lizards at a survey site declined by almost 80%. It is unknown to what extent the vegetation removal may have caused direct mortality or whether some of these lizards could have moved to adjacent sites; however, we never found these lizards in nearby areas and in only one case was a site recolonized after vegetation grew back. Recolonization of a site is rare because roads and buildings can act as barriers to movement within town leading to reduced dispersal (Wall, 2014). We suggest that declines at our survey sites have occurred in large part because once shrubs were removed the habitat would have effectively been the “open” microhabitat and the temperatures at ground level would often exceed their CT_max_ during the summer months.

Vegetation removal may also influence horned lizard prey populations by degrading their thermal environment, reducing foraging opportunities, or changing predation levels and so could contribute to the decline of lizards. In Kenedy and Karnes City, Texas horned lizards eat mostly smaller ants (*Pheidole* spp., 40% of diet) and harvester termites (*Tenuirostritermes cinereus*, 34% of diet), while harvester ants only make up 8% of their diet (Alenius, 2018). *Tenuirostritermes cinereus* exhibits diurnal open‐air foraging that is constrained by hot temperatures and low humidity levels. This species is usually found foraging in overcast and humid conditions, such as under vegetation or during the early morning hours (Alenius, 2018; Nutting et al., 1974; Scheffrahn & Rust, 1983). Ants are also sensitive to increased midday temperatures and have a bimodal pattern of activity (Whitford et al., 1980, 1981; Whitford & Bryant, 1979). Vegetation provides shade and a humid microenvironment, and therefore could increase foraging time for horned lizards.

Open microhabitats (i.e., bare ground) are still important for thermoregulation during the morning and evening hours to reach adequate *T*_*b*_. Burrowing in the middle of the day can potentially reduce temperatures below CT_max_, although burrowing in open areas such as the locations we placed the models would result in a *T*_*e*_ that was often above their upper *T*_set75_ for about 6 h of the day. It is not clear how often burrowing behavior may be related to thermoregulation since they only bury themselves a few centimeters under the surface of loose dirt or sand and they often burrow while under vegetation (Burrow et al., 2001; Whitford & Bryant, 1979). Burrowing is effective at making the lizards invisible, so it may more often function as a predator avoidance strategy when lizards are inactive. Texas horned lizards will also climb onto the trunk or lower branches of shrubs during the hottest times of the day for thermoregulation (Burrow et al., 2001; Whitford & Bryant, 1979). We have never observed this behavior in Kenedy or Karnes City, so we did not place models in those areas; however, studies of this species in other areas should include models in shrubs to evaluate their daily temperature profiles.

Preferred body temperature at our study sites (*T*_set_ = 35.7 ± 0.33°C) was in between reported *T*_set_ from other studies of Texas horned lizards (Table 1), but close to the average *T*_set_ of 20 species of *Phrynosomatids* (35.1 ± 2.2°C; Clusella‐Trullas & Chown, 2014). Field body temperature (*T*_*b*_ = 33.6 ± 0.30°C) was similar to one reported value (33.4 ± 0.45°C; Lara‐Reséndiz, Arenas‐Moreno, et al., 2015) and lower than three other reported body temperatures for Texas horned lizards (35.7 ± ND °C, Brattstrom, 1965; 37.3 ± 0.30°C, Pianka & Parker, 1975; 35.2 ± 3.44 SD °C, Russell, 2001). Nonetheless, *T*_*b*_ is lower than the mean environmental temperatures available to them (*T*_*e*_ = 35.2 ± 1.1°C) but falls within the lower *T*_set25_ range for lizards at our study sites. Horned lizards at our field sites thermoregulated with lower effectiveness (*E* = 0.31), which is consistent with less precision and relaxed thermoregulation found across *Phrynosoma* spp. (Pianka & Parker, 1975). Thermoregulatory effectiveness, as measured by ${\bar{d}}_{e}–{\bar{d}}_{b}$, was 0.71, which also indicates some thermoconforming behavior and a thermally benign environment. Horned lizards at our site could be keeping their *T*_*b*_ lower because precise thermoregulation is less important than other activities. For example, foraging could potentially be prolonged in shaded areas with lower temperatures.

There was a negative correlation between the average thermal quality (*d*_*e*_) at a site and average density (lizards/hectare) of horned lizards. All lizards disappeared from the three sites regularly surveyed in Kenedy by 2019. The thermal data collected from these three sites and two others that historically had horned lizards revealed that these sites had lower thermal quality than sites in Karnes City. The lower thermal quality of these sites coupled with vegetation removal probably contributed to the disappearance of lizards at these sites. The lower thermal quality in Kenedy is in part related to the configuration of habitat. All of the sites were in open fields, while many of the sites in Karnes City were alleyways or had alleyways associated with an open field. Alleyways had significantly higher thermal quality than fields and higher densities of lizards compared to the fields. Alleyways in these towns consist of dense vegetation (e.g., shrubs and grasses) along fence lines and a variable canopy cover with a dirt road in the middle. This configuration allows lizards to sun and forage, then retreat into the nearby vegetation when temperatures increase. Fields have isolated shrubs, trees with bushes, or brush piles that are relatively separated from each other. Alleyways represent a configuration of thermal refugia that are more dispersed with a gradient of temperatures near each other, whereas fields have a more clumped distribution of thermal refugia, and so are less favorable because of the energetic costs of moving between distant clusters of favorable microhabitats to maintain body temperature (Huey & Slatkin, 1976; Sears et al., 2016; Thompson et al., 2018).

Texas horned lizards may be well suited to living in some types of human modified habitats that result in a heterogeneous mix of microhabitats. A recent meta‐analysis of reptile responses to anthropogenic habitat modification found that the family *Phrynosomatidae* had a less negative response to human habitat modification than other groups of lizards, suggesting they may be adapted to more disturbed habitats or habitats that have features of disturbance such as arid lands with sparse vegetation (Doherty et al., 2020). Towns like Kenedy and Karnes City can provide small reptiles like horned lizards with a heterogeneous mix of closely spaced microhabitats through effective landscaping. Within town, Texas horned lizards utilize areas that contain native grasses, have some bare ground, and are mowed or trimmed regularly (Wall, 2014). The lizards use both non‐native and native shrubbery, large prickly pear cacti (*Opuntia* spp.), brush piles, the inside of old sheds, and under pier and beam houses as thermal refugia and hiding places. Texas horned lizards in these towns will require maintaining this diverse mix of closely spaced microhabitats, especially thermal refugia. Conserving and creating thermal refugia is likely to be one of the most important and practical conservation actions that can be taken to help small ectotherms persist in human modified landscapes and cope with increasing temperatures due to climate change (Attum et al., 2013; Gaudenti et al., 2021; Grimm‐Seyfarth et al., 2017; Ivey et al., 2020; Kearney et al., 2009; Suggitt et al., 2018).

## AUTHOR CONTRIBUTIONS

**Mary R. Tucker:** Conceptualization (lead); data curation (lead); formal analysis (lead); funding acquisition (equal); investigation (equal); methodology (lead); project administration (equal); resources (equal); software (lead); supervision (equal); validation (equal); visualization (lead); writing – original draft (lead); writing – review and editing (equal). **Daniella Biffi:** Conceptualization (equal); data curation (equal); formal analysis (equal); funding acquisition (lead); investigation (equal); methodology (equal); project administration (equal); resources (equal); software (equal); supervision (equal); validation (equal); visualization (supporting); writing – original draft (supporting); writing – review and editing (supporting). **Dean A. Williams:** Conceptualization (lead); data curation (equal); formal analysis (equal); funding acquisition (equal); investigation (equal); methodology (equal); project administration (equal); resources (equal); software (equal); supervision (lead); validation (equal); visualization (equal); writing – original draft (equal); writing – review and editing (equal).

## FUNDING INFORMATION

This project was funded by grants from the Andrews Institute of Mathematics & Science Education at TCU, TCU Research and Creative Activities Fund, Science & Engineering Research Center, and TCU Invests in Scholarship Fund.

## CONFLICT OF INTEREST STATEMENT

None declared.

## Data Availability

The data used in the analysis of this paper can be found in the Dryad Data Repository: https://doi.org/10.5061/dryad.z8w9ghxj5.

## Appendix

**TABLE A1:** Generalized linear model (GLM) selection for analysis of field body temperatures (*T*_*b*_) for Texas horned lizards from 2019 to 2021 using bias‐corrected Akaike's information criterion (AIC_c_). *k* is the number of parameters in the model. ΔAIC_c_ is the difference between the best model and the comparison model. Akaike weights (*w*_*i*_) calculates the relative likelihood of each model being the best‐fitting model among the set of candidates.

Model	*k*	AIC_c_	ΔAIC_c_	*w*_*i*_
*T*_*b*_ ~ Year + Month + Age + Sex + BC + Time + Sex*BC	7	747.34	8.65	0.006
*T*_*b*_ ~ Year + Age + Sex + BC + Time + Sex*BC	6	742.99	4.30	0.054
*T*_*b*_ ~ Year + Age + Sex + BC + Time	5	741.14	2.45	0.136
*T*_*b*_ ~ Year + Age + Sex + Time	4	739.32	0.63	0.339
*T*_*b*_ ~ Year + Age + Time	3	738.69	0	0.054

**TABLE A2:** Generalized linear model (GLM) selection for analysis of field body temperatures (*T*_*b*_) for Texas horned lizards from 2020 to 2021 using bias‐corrected Akaike's information criterion (AIC_c_). Microhabitat data was not collected during the 2019 field season. *k* is the number of parameters in the model. ΔAIC_c_ is the difference between the best model and the comparison model. Akaike weights (*w*_*i*_) calculates the relative likelihood of each model being the best‐fitting model among the set of candidates.

Model	*k*	AIC_c_	ΔAIC_c_	*w*_*i*_
*T*_*b*_ ~ Year + Month + Age + Sex + BC + Time + Microhabitat + Sex*BC + Microhabitat*BC	9	475.81	17.29	0.0001
*T*_*b*_ ~ Year + Age + Sex + BC + Time + Microhabitat + Sex*BC + Microhabitat*BC	8	451.96	11.44	0.0020
*T*_*b*_ ~ Year + Age + Sex + BC + Time + Microhabitat + Sex*BC	7	447.18	6.66	0.0214
*T*_*b*_ ~ Year + Age + Sex + BC + Time + Microhabitat	6	444.54	4.02	0.0802
*T*_*b*_ ~ Year + Age + Sex + Time + Microhabitat	5	441.92	1.4	0.2974
*T*_*b*_ ~ Year + Sex + Time + Microhabitat	4	440.52	0	0.5989

**TABLE A3:** Generalized linear mixed model (GLMM) selection for analysis of preferred body temperatures (*T*_set_) for Texas horned lizards for 2021 using bias‐corrected Akaike's information criterion (AIC_c_). *k* is the number of parameters in the model. ΔAIC_c_ is the difference between the best model and the comparison model. Individual horned lizard is set as a random effect and month, time as fixed effects, and gradient quadrant temperatures (in order from closest to heat source to furthest: front, first quarter, second quarter, and back) as covariates. Akaike weights (*w*_*i*_) calculates the relative likelihood of each model being the best‐fitting model among the set of candidates.

Model	*k*	AIC_c_	ΔAIC_c_	*w*_*i*_
*T*_set_ ~ Individual HL + Month + Time + Front + First Quarter + Second Quarter + Back	7	587.22	28.71	5.83 × 10^−7^
*T*_set_ ~ Individual HL + Month + Time + First Quarter	4	587.97	29.46	4.01 × 10^−7^
*T*_set_ ~ Individual HL + Month + Time	3	590.53	32.02	1.11 × 10^−7^
*T*_set_ ~ Individual HL + Month + Time(Month)	3	558.51	0	1.00
